# Evaluation of the Anti-Shigellosis Activity of Dietary Isothiocyanates in *Galleria mellonella* Larvae

**DOI:** 10.3390/nu13113967

**Published:** 2021-11-07

**Authors:** Dariusz Nowicki, Klaudyna Krause, Monika Karczewska, Agnieszka Szalewska-Pałasz

**Affiliations:** Department of Bacterial Molecular Genetics, Faculty of Biology, University of Gdansk, Wita Stwosza 59, 80-308 Gdansk, Poland; dariusz.nowicki@ug.edu.pl (D.N.); klaudyna.krause@phdstud.ug.edu.pl (K.K.); monika.szalkowska@phdstud.ug.edu.pl (M.K.)

**Keywords:** *Shigella dysenteriae*, isothiocyanates, sulforaphane, phenethyl isothiocyanate, stringent response, *Galleria mellonella*

## Abstract

Cruciferous vegetables, widely present in daily diets, are a rich source of organosulfur compounds with proven health benefits, especially chemopreventive or antioxidative effects. Isothiocyanate derivatives (ITCs) exhibit a broad spectrum of biological and pharmacological activity and recently, their antibacterial properties have been of particular importance. Here, we have focused on the anti-shigellosis activity of sulforaphane (SFN) and phenethyl ITC (PEITC). The genus *Shigella* causes gastroenteritis in humans, which constitutes a threat to public health. Production of a potent Stx toxin by *S. dysenteriae* type 1 results not only in more severe symptoms but also in serious sequela, including the hemolytic uremic syndrome. Here, we present evidence that two aliphatic and aromatic ITCs derivatives, SFN and PEITC, have an effective antibacterial potency against *S. dysenteriae,* also negatively regulating the *stx* gene expression. The molecular mechanism of this effect involves induction of the global stress-induced stringent response. ITCs also inhibit bacterial virulence against the Vero and HeLa cells. We present evidence for the therapeutic effect of sulforaphane and phenethyl ITC against a *S. dysenteriae* infection in the *Galleria mellonella* larvae model. Thus, our results indicate that isothiocyanates can be effectively used to combat dangerous bacterial infections.

## 1. Introduction

Natural products and their derivatives are an important source of bioactive compounds and represent more than one-third of all FDA-approved new molecules to date [1]. Among natural products, isothiocyanates (ITCs) are one of the major secondary metabolites that occur naturally and ubiquitously in plants. The dietary sources of ITCs are mainly vegetables, sprouts and seeds from the Brassicaceae family. These volatile substances are formed as a result of the substrate hydrolysis of glucosinolates, by the enzymatic activity of myrosinase in plant tissue for the protection against pathogens and animal-related damages [2]. Currently, it is well established that ITCs exhibit diverse biological activities, such as chemopreventive, anti-inflammatory and anticancer effects, and their role in combating bacterial infection has recently attracted increasing attention, making this class of molecules a potential source of new compounds for industry and medicine [3,4,5,6,7].

The *Shigella* genus is an etiological factor of bacterial diarrhea and dysentery worldwide, especially in the developing countries [8]. *Shigella* is considered a frequent bacterial causative agent in the diarrhoea of travellers visiting low-income areas. Apart from foreign travel, risk factors for shigellosis in industrialized countries include children who are <5 years or subjects in day-care centres. A dose as low as 10–100 bacteria is sufficient to cause an infection [9]. This high risk of infection ensures that the total number of shigellosis episodes remains constant, despite reduction in shigellosis-related deaths in the healthcare system in the past two decades [9,10]. The endemic outbreaks of shigellosis emerge even more often in developed countries these days [11]. Shigellosis can be caused by four main Shigella species: *S. sonnei, S. flexneri, S. boydii* and *S. dysenteriae* [8].

In addition to various virulence factors harbored by the representatives of this genus, serotype 1 of *S. dysenteriae* can produce a potent toxin that inhibits protein synthesis. The first report on the production of this toxin, called the Shiga toxin, dates from the late nineteenth century (1898), when Kiyoshi Shiga described cases of bloody diarrhoea [12]. Dysentery outbreaks caused by *S. dysenteriae* type 1 occurred several times in the 20th century, causing a serious threat to public health. Infections with *S. dysentieriae* result in a variety of symptoms, ranging from mild diarrhoea to hemorrhagic colitis, and are due to bacteria invading the mucosal surface of the intestines [13]. In some patients, the effect of toxin production during *S. dysenteriae* infection can lead to serious complications and sequela, including neurological problems and the hemolytic uremic syndrome caused by invasion of the vascular endothelium and subsequent lesions [14,15]. The Shiga toxin, responsible for the virulence of *S. dysenteriae* type 1, is encoded on the bacterial chromosome, although presence of bacteriophage-derived genes has been reported. However, the toxin production is not related to prophage excision and induction, contrary to enterohemorrhagic *E. coli* strains [16,17]. Due to extensive knowledge of *Shigella* virulence and genetics, representatives of this genus make good models for bacterial invasive pathogenesis [18].

As mentioned above, the Shiga toxins can be also harbored by *Escherichia coli* strains, resulting in their toxicity. In *E. coli*, the production of Shiga toxin is dependent on the induction of the lambdoid prophage, as the *stx* genes are under the control of this phage’s late promoter. As the induction is mediated by the SOS response, DNA damage and other stresses, such as free radicals, can lead to phage induction and toxin release, which means that the use of standard antibiotics is discouraged in the course of EHEC infection. In our previous work, we showed that plant secondary metabolites, isothiocyanates (ITCs), effectively inhibit the growth of enterohaemorrhagic *E. coli* (EHEC) strains [19,20]. We showed that the induction of phage lytic development and the Stx toxin production was also inhibited by the action of ITC [19,20]. Moreover, the toxicity of bacterial lysates treated with ITC against human and simian cell cultures was significantly reduced [19,20]. The molecular mechanism underlying the antibacterial effect of phytochemicals involves the global stress response, the stringent response. Its alarmones, ppGpp and pppGpp, accumulate during various stress conditions and nutrient limitation, leading to remodeling of the gene expression pattern by direct and indirect action on the transcription machinery [21].

The stringent response is widespread among bacteria and both *E. coli* and *S. dysenteriae*, belonging to the Enterobacteriaceae family, can synthesize (p)ppGpp via two synthetases, RelA and SpoT. However, previously we presented evidence that the antibacterial effect of ITC is not always related to the stringent response induction, e.g., in *Bacillus subtilis*, and could be based on the disruption of the integrity of the cellular membrane integrity [22]. Nevertheless, we showed that ITC could inhibit the growth of several bacterial pathogens, including *Klebsiella pneumoniae* and *Staphylococcus aureus*. Recently, we also presented evidence for the antibacterial action of ITCs against another toxin-producing bacterium, *Vibrio cholerae* [23].

Developing a new possible line of infection treatment is important, especially nowadays, when multidrug-resistant bacterial strains emerge and when designing new antibiotics is a long process. In this work, we ask whether the growth of a human pathogen bearing the Stx encoding genes, *S. dysenteriae*, is affected by two representative ITC, sulforaphane (SFN) and phenethyl ITC (PEITC). We show that these two compounds can effectively inhibit bacterial growth, toxin gene expression and toxicity against eukaryotic cells. Furthermore, we presented the SFN efficient antishigellosis effect in the surrogate *Galleria mellonella* larvae model based on the enhanced phagocytosis of bacterial cells.

## 2. Materials and Methods

### 2.1. Bacterial Strains and Growth Conditions

The following strains were used in this study: *S. dysenteriae* SZ2855/97 Japan, *S. dysenteriae* Bonn/76, *S. dysenteriae* 156-36/88 Osaka, kindly provided by Dr. Anselm Lehmacher, *S. flexneri* ATCC 12022, *S. sonnei* ATCC 25931. Bacteria were typically grown in Lysogeny Broth (Lennox, Sigma-Aldrich, Darmstadt, Germany) at 37 °C with orbital shaking at 160 rpm.

### 2.2. Bacterial Growth Inhibition

To assess the minimum inhibitory and bactericidal concentrations (MIC/MBC) of the tested compounds, the twofold broth microdilution method was used according to that BSAC standard, with the inoculum of 5 × 10^5^ cells/well in 96 well plates [22]. The concentration of ITC ranged from 10–0.0125 mM. The results were obtained using the EnSpire (Perkin Elmer Singapore Pte. Ltd, Singapore) instrument after 20 h incubation at 37 °C. MBC was assessed in the same procedure but after 20 h samples were serially diluted in PBS and spotted (10 µL) on solid agar LB plates. Then, a concentration of ITC that kills 99.9% of cells was determined after over-night incubation at 37 °C. The kinetics of growth inhibition were measured spectrophotometrically (A_600_) in the presence of relevant concentrations of ITCs.

### 2.3. Assessment of (p)ppGpp Accumulation in Bacteria

Cellular levels of the stringent response alarmones, i.e., ppGpp and pppGpp, were measured basically as described previously [24], with modifications. The overnight bacterial culture was grown in the MOPS (4-morpholinepropanesulfonic acid) minimal medium and then was diluted in the same medium, but with a low phosphate concentration (0.4 mM) and then cultivated to A_600_ of 0.2. Then, the cultures were diluted in a 1:10 ratio in the same medium, but with addition of [^32^P]orthophosphoric acid (150 μCi/mL) and grown for two generations. Next, at time zero, ITCs at 1 × MIC, or serine hydroxamate (SHX), at 1 mM was added. At specific times, samples were collected and lysed with formic acid (13 M) in three cycles of the freeze-thaw procedure. Following centrifugation, nucleotides were separated by thin-layer chromatography on PEI cellulose plates (Sigma-Aldrich, Darmstadt, Germany) in 1.5 M potassium phosphate buffer (pH 3.4). The chromatograms were analyzed using the Typhoon 9200 Phosphorimager (GE Healthcare, Uppsala, Sweden). QuantityOne (BioRad, Hercules, CA, USA) software was used for densitometry analysis.

### 2.4. RNA Isolation and RT-PCR Analysis

Bacteria were grown to OD_600_ = 0.1 (t = 0) in LB and treated with SFN doses equal to 1 × MIC, ¼ × MIC and 1/16 × MIC, or SHX (1 mM), mitomycin C (0.5 µg/mL); for negative control untreated culture was used. Samples for RNA purification were collected 180 min after treatment. Total RNA was extracted in duplicate from two biological replicate cultures of each strain using the RNeasy MINI kit (Qiagen GmbH, Hilden, Germany) according to the manufacturer’s protocol. EvoScript Reverse transcriptase (Roche, Basel, Switzerland) and random primers were used to create cDNA from RNA samples. Quantitative real-time PCR (qRT-PCR) was performed using LightCycler 480 (Roche Diagnostics AG, Rotkreuz, Switzerland) and the following primers: stx1—CGATGTTACGGTTTGTTACTGTGACAGC; stx1R—AATGCCACGCTTCCCAGAATTG; recAF—CTGGAAATCTGTGACGCCCT; and recAR—ATCGCCTGGCTCATCATACG. All data were normalized to levels of *recA*, and analyzed using the comparative cycle threshold (CT) method. Target gene expression levels were compared by the relative quantification method 2^−ΔΔCT^ [25].

### 2.5. Assessment of Toxicity of Bacterial Lysates on the HeLa and Vero Cells

The procedure was performed basically as described [19]. Human HeLa or simian Vero cells were grown in the DMEM medium (Gibco, Waltham, MA, USA), pH 7.4, supplemented with 10% heat-inactivated fetal bovine serum (FBS) and 1 × Antibiotic and Antimycotic solution (all purchased from Sigma, Darmstadt, Germany) and incubated at 37 °C in a humidified 5% CO_2_ atmosphere. To evaluate bacterial toxicity towards the HeLa or Vero cells, bacterial lysates were prepared. Overnight cultures of *S. dysenteriae* were grown in LB medium. After 100-fold dilution in fresh MOPS minimal medium (containing 0.5% glucose), the cultivation was continued until A_600_ reached 0.1. Then, mitomycin C, ITC, and/or SHX were added to the cultures to various final concentrations and cultures were incubated with shaking for 30 min. To prepare cell-free lysates, cultures were centrifuged and lysates were filtered using PVDF syringe filters (0.22 mm; Roth). Lysates were then serially diluted in DMEM. Cytotoxicity was assessed using the 3-(4,5-dimethyl-2-thiazolyl)-2,5-diphenyl-2H-tetrazolium bromide (MTT) assay, performed as described previously [26]. HeLa or Vero cell lines were seeded at 1 × 10^4^ cells/well in 96-well plates and incubated overnight. Then, the growth medium was substituted with fresh medium supplemented with the tested compounds (5 µL of lysates were added to 95 mL of DMEM). After 48 h of incubation, the medium was changed to the MTT solution (100 µL) (MTT was diluted in PBS to 1 mg/mL). Plates were incubated at 37 °C for 4 h, and then the medium was removed. The purple formazan product was dissolved in 100 µL dimethyl sulfoxide (DMSO) and quantified by measuring A_570_. The data are presented as means (with SD) from at least three independent experiments, relative to nontreated cultures.

### 2.6. Study of Pathogenicity of S. dysenteriae by Employing the GALLERIA Mellonella Larvae

The larvae of *Galleria mellonella* (the greater wax moth), TruLarv, were obtained from BioSystems Technology (Credition, UK). Larvae were stored in the darkness at room temperature before experiments. The infection was conducted basically, as described previously [27]. Briefly, the healthy larvae of 250–300 mg weight with no melanization symptoms were selected and transferred to the Petri dish. The larvae (*n* = 15) were surface sterilized with ethanol before each injection procedure. Insects were infected with the varying number (from 10^2^ to 10^8^ c.f.u./larva) of *S. dysenteriae* cells using a 100 µL Hamilton syringe into the last left foreleg with a blunt tip needle. The control insects were injected with PBS. Caterpillars were examined every 24 h (up to 96 h) for changes in behavior and melanization; the number of living larvae was assessed. For the treatment experiment, SFN (25 mg/kg) and/or azithromycin (6.25 mg/kg) as a standard antibiotic used against gastrointestinal infection (the dosage corresponds to human treatment) were injected 1 h after infection. Three independent experiments were performed.

### 2.7. In Vivo Phagocytosis Assay

The heat-killed and FITC labelled *S. dysenteriae* SZ2855/97 Japan bacteria were prepared as described [28]. Briefly, the 10^9^/mL cells were heated to 70 °C for 1 h, washed twice and incubated in 0.5% carbonate buffer (pH 9.5) containing FITC (0.15 mg/mL) for 30 min at 37 °C. Larvae (*n* = 10) were treated with SFN (25 mg/kg) or PBS as control and after 30 min were challenged with 10 µL of labelled bacteria injected into the larvae’s last left foreleg. 3 h after infection, 5 larvae from each group were bled into Eppendorf tubes with heparin and trypan blue (1% in PBS). Samples (5 µL) were placed on the coverslips and visualized under a Leica DMI4000B microscope fitted with a DFC365FX camera (Leica Microsystems, Wetzlar, Germany). The phagocytosis rates were assessed from at least 5 independent fields (100 hemocytes) for each sample.

## 3. Results and Discussion

### 3.1. ITC Inhibit S. dysenteriae Growth and SHIGA Toxin Production

A desirable antibacterial compound must be safe for therapy, should inhibit bacterial growth and development and also preferably inhibit virulence factor secretion. Importantly, to avoid unnecessary burden for the patient’s organism, the lowest possible effective concentration should be used. Thus, a first step to assess the antimicrobial potential of a given compound is determination of the minimum inhibitory concentration (MIC). Three different isolates of *S. dysenteriae* were used for the assessment of the ITC effect. For this work, we chose SFN and phenethyl PEITC as these ITCs have been quite well studied for their effects in humans. Moreover, PEITC was shown to be a potent antibacterial agent against *E. coli* and *S. aureus* as reported in [29], and also against EHEC strains [19]. The antibacterial effect of SFN and other aliphatic ITC was also reported [22,30] and furthermore, as part of the daily diet SFN is known for its chemopreventive and anticancer effect [31,32]. The determination of MIC (Table 1) indicates that both ITCs exert their effect against all tested *S. dysenteriae* isolates, as well as against *S. sonnei* and *S. flexneri*.

**Figure 1:**
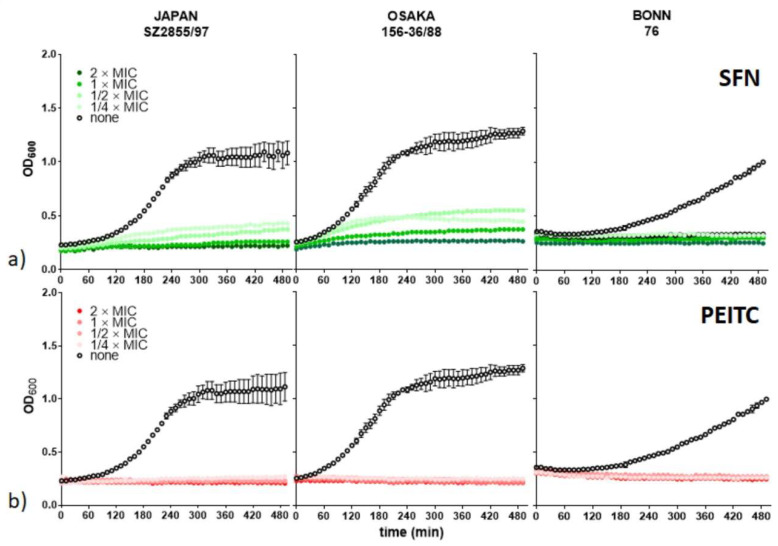
SFN and PEITC affect bacterial growth. *S. dysenteriae* isolates (indicated above graphs) were cultured in the presence of (**a**) SFN and (**b**) PEITC at indicated concentrations related to the MIC determined previously (Table 1).

The minimum inhibitory concentrations are relatively low for S. *dysenteriae* strains (in the range between 11–44 and 41–81 µg/mL, for SFN and PEITC, respectively), which enhances the possibility of employing these ITC in the therapy of *Shigella* infection. Notably, in comparison to the MIC for EHEC strains [19,20], the growth of *S. dysenteriae* representatives was impaired at the same or lower range of compounds. Nevertheless, the other two *Shigella* representatives, *S. sonnei* and *S. flexneri*, were less sensitive to the ITCs treatment with their MIC values greater than 100 µg/mL, and therefore we decided to exclude them from further analysis. Next, we assessed growth of *S. dysenteriae* in the presence of varying concentrations of the ITCs tested, and found that even at concentrations of ¼ MIC, the ITCs were able to significantly inhibit growth (*p* <0.001) of bacterial cultures in a rich liquid medium (Figure 1). Interestingly, the PEITC antibacterial effect does not appear to be dose-dependent, as complete growth inhibition was observed for all concentrations tested and for all strains. We observed *S. dysenteriae* Boon/76 was the most sensitive to SFN. Thus, we can conclude that both SFN and PEITC act as effective antibacterial agents against *Shigella* strains, providing a promising course of further studies.

### 3.2. ITCs Reduce the Toxicity of S. dysenteriae

**Figure 2:**
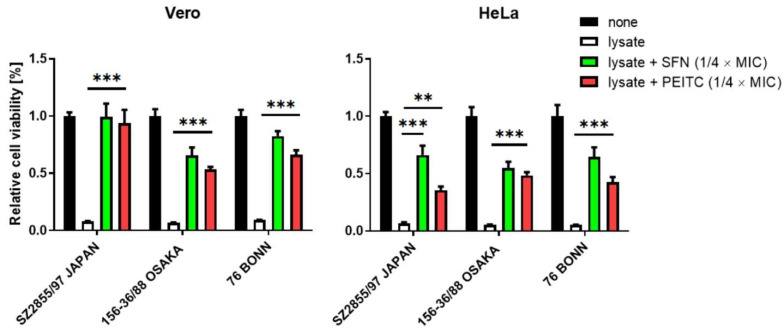
ITC reduces the toxicity of *S. dysenteriae* lysates. The effect of SFN on viability of HeLa and Vero cells treated with *S. dysenteriae* lysate was assessed after 74 h in the absence or presence of SFN at concentrations indicated. Untreated culture was used as a negative control (black bar) and a culture treated with bacterial lysate was used as a positive control (white bar). SFN (green bar) and PEITC (red bar) treated groups were assessed against positive control using Student’s *t*-test. Statistical significance is marked with asterisks: *** *p* <0.001; ** *p* <0.01.

The most harmful effect during *S. dysenteriae* infection is caused by production of the Shiga toxin, and thus not only growth inhibition but also toxin synthesis downregulation would be expected from a potential antimicrobial compound. Since the eukaryotic cell lines: human HeLa and simian Vero are sensitive to Shiga toxin [33], they can serve as good indicators for bacterial virulence. Thus, we tested viability of these cells when exposed to the ITC-treated or untreated bacterial lysates. We observed that the treatment of *S. dysenteriae* strains with PEITC and SFN at the concentrations of ¼ MIC (which effectively impaired bacterial growth) resulted in a statistically significant decrease in toxicity of bacterial lysates, as tested by the MTT assay (Figure 2). Alleviation of bacterial toxicity was similar for both ITCs tested. The results are in concert with those previously reported by us for the EHEC strain, where PEITC-treated cultures showed a notably lower virulence against the Vero and HeLa cells [19].

Here, we show that downregulation of Shiga toxin production by ITC (as shown in Figure 2) has a direct effect on increased cell viability. Moreover, there are no defects in cell morphology when tested eukaryotic cells are exposed to the lysates of bacterial cells treated with ITC, contrary to cells exposed to untreated bacterial lysates. It is worth mentioning that, as we showed previously [19] and as was already reported by others [31], when PEITC and SFN are used alone at the concentrations employed for this study, they do not have a detrimental effect on the cell lines’ viability.

### 3.3. Induction of the Stringent Response Underlines the Antibacterial Effect of ITC on S. dysenteriae

**Figure 3:**
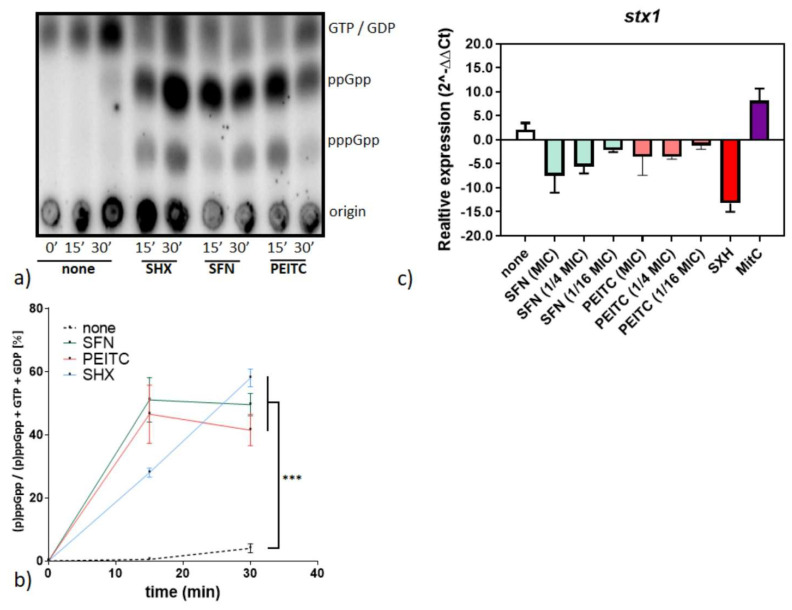
SFN and PEITC impair expression of the Shiga toxin genes and induce stringent response in *S. dysenterae*. (**a**) Synthesis of the stringent response alarmones, ppGpp and pppGpp, was assessed by culturing bacteria with the addition of [^32^P]orthophosphoric acid (150 μCi/mL) followed by cell lysis and nucleotide separation by thin-layer chromatography. ITC or SHX were added at time zero. Samples were withdrawn at 15 and 30 min after the compound addition. The positions of ppGpp and pppGpp are indicated at the right side of the panel. (**b**) Relative cellular level of (p)ppGpp was assessed densitometricaly, where treated samples were compared to the nontreated negative control (at the 30 min endpoint) using Student’s *t*-test. Statistical significance is marked with asterisks: *** *p* < 0.001; (**c**) Relative expression of *stx*1 in *S. dysenteriae* SZ2855/97 Japan was determined by qRT-PCR and is presented using 2^−ΔΔCT^ quantification normalized to the *recA* gene expression level.

The observed growth inhibition of *Shigella* strains in the presence of ITC led us to search for the mechanisms underlying this process. As we know from our previous studies, in *E. coli* ITCs exerted its effects via induction of the stringent response. Therefore, we evaluated cellular levels of the stringent response alarmones, ppGpp and pppGpp, in the *S. dysenteriae* SZ2855/97 Japan strain treated with PEITC, SFN, or serine hydroxamate, the amino acid analog that serves as a control of the stringent response induction. We also compared the observed nucleotide levels visualized by thin-layer chromatography with an untreated control (Figure 3a,b). The strong signal of both tetra- and pentaphosphate is visible in the SHX control, indicating that bacteria react to amino acid starvation by alarmone accumulation. Interestingly, both SFN and PEITC, induced the stress alarmone accumulation to an extent comparable to that induced by SHX. Apparently, similar to many bacterial strains tested so far [22], the basis of the effect of ITC-mediated growth inhibition is induction of the global stress response. The stringent response, as a general stress mechanism, is widely conserved in the Enterobacteriaceae family and *Shigella sp*. genus. The *relA*/*spoT* genes display high sequence similarity to their *E. coli* homologues [34]. Accumulation of (p)ppGpp causes reprogramming of bacterial gene expression, shutting down such energy-consuming processes as ribosomal component synthesis, and, in general, expression of numerous household genes, while expression of genes involved in the stress response and those recognized by alternative sigma factors is activated. Such a global alteration in bacterial cellular metabolism results in growth inhibition, and importantly, leads to the downregulation of the Shiga toxin production (directly or indirectly). Another mode of action of ITCs can also involve destabilization of cellular membrane integrity, as we observed in our recent work [22] and as was suggested previously [29]. However, this phenomenon in *B. subtilis* was not triggered by the stringent response induction. Thus, it can be concluded from the data presented here that the major mechanism underlying growth inhibition of *S. dysenteriae* involves the alarmone accumulation. It was also suggested that ppGpp and its cofactor DksA play a certain role in *Shigella* virulence [35,36], and interestingly, concentrations of the alarmones during the onset of the stringent response induced by ITC significantly exceed those observed during normal growth. Thus, the effect of (p)ppGpp accumulation mediated by ITCs is growth inhibition, which provides a promising course for additional investigation of ITCs application.

Since the pathogenicity of *Shigella* strains is strictly related to the synthesis of Stx toxin, we tested expression of the *stx* gene in *S. dysenteriae* SZ2855/97 Japan by qRT-PCR after the SFN/PEITC treatment (Figure 3c). We assessed the effect of both compounds at 1 × MIC and ¼ MIC, and found that they effectively downregulated the level of *stx* gene expression. Namely, the fold change observed for SFN treatment was 0.16 ± 0.01, 0.18 ± 0.04, while for PEITC it was 0.22 ± 0.11, 0.28 ± 0.03 for 1 × MIC and ¼ MIC, respectively. However, even lower concentrations of SFN/PEITC (1/16 × MIC) also had an inhibiting effect (0.47 ± 0.09 and 0.83 ± 0.79, fold level change for SFN and PEITC, respectively), although it was not as pronounced. On the other hand, mitomycin C, the SOS triggering factor, caused a significant increase in the *stx* gene transcription (Figure 3c). The latter effect is caused by the general stress exerted by mitomycin C, as it was reported that addition of this drug had caused an increase in production and secretion of Shiga toxins through the outer membrane vesicles [37]. Although the Shiga toxin production by *S. dysenteriae* was reported not to be directly related to the prophage induction, the presence of genes belonging to a defective prophage in the vicinity of *stx* genes may have an influence on the toxin gene expression. Furthermore, the *stx* encoding region within this genus is remarkably conserved [38]. Our results indicate that ITCs do not only inhibit the growth of *Shigella* strains, but more importantly, reduce expression of the Shiga toxin genes.

### 3.4. SFN Reduces the Toxicity of S. dysenteriae In Vivo

Our cell lines experiments described above provided further proof for potential therapeutic use of ITC in treating *Shigella* infections. However, more important are studies performed in living organisms, not only in isolated cell cultures. Thus, we decided to employ an animal model. The *Shigella* infections have been studied in many models, including primates, rodents and piglets [18]; nevertheless, the use of these animals is expensive, requires a specific facility and raises significant ethical issues.

**Figure 4:**
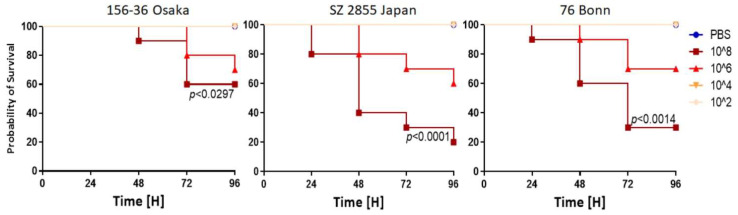
Survival analysis of *G. mellonella* challenged with *S. dysenteriae* strains. Larvae (*n* = 15) were injected with (10 µL) PBS (blue circle) or *S. dysenteriae* suspension in PBS in the range of 10^2^ to 10^6^ c.f.u./per dose. Long rank Mantel-Cox statistic test was used to assess differences between infection groups and the PBS-treated negative control group. At least three independent experiments were conducted, and a representative one is presented on the plot.

Thus, a more convenient model was introduced, namely, the larvae of *Galleria mellonella*, the greater wax moth. This surrogate model is widely used for novel antibiotics’ efficacy studies, pathogen virulence and resistance screening, and its use in monitoring *Shigella* infections has been described in detail [27]. Briefly, these insects are easy to maintain and handle, their size allows for injection of pathogenic bacteria and simple-to-monitor steps of infection were described. Furthermore, the insect immune system, although evolutionarily distant, presents significant similarities to vertebrates, which makes it useful to make conclusions about general response to pathogens. In particular, specific larvae cells, hemocytes, provide a line of defense against pathogenic bacteria due to phagocytosis [39]. In addition, the activation of hemocytes provides a signal for melanization in larvae [40]. Thus, the development of the infection can be monitored by melanization, with an easy-to-determine scale [27]. Therefore, we took advantage of the *G. mellonella* model and assessed the effect of SFN on the progress of infection. First, the survival of the larvae was monitored after injection of various c.f.u. of the three tested *S. dysenteriae* isolates. As shown in Figure 4, the most virulent effect was caused by the *S. dysenteriae* SZ2855/97 Japan strain, with the lowest survival level (down to 20% after 96 h) at a dose of 10^8^ per ml of bacteria. Thus, for the ITC test, this inoculum was chosen.

**Figure 5:**
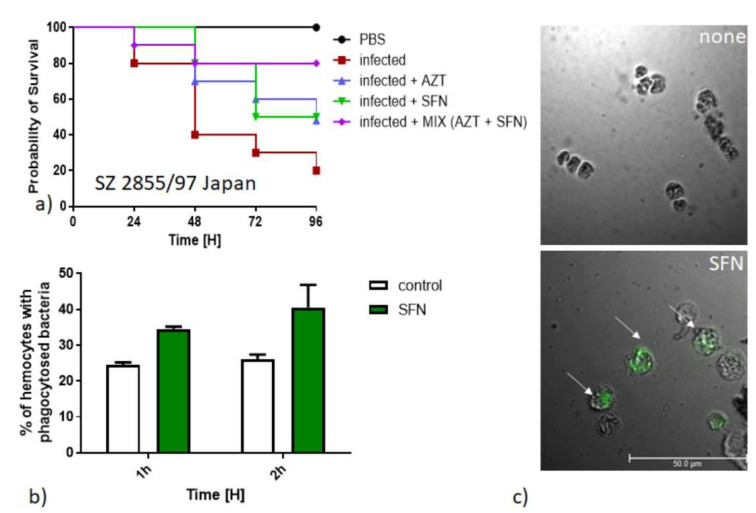
SFN inhibits virulence of *S. dysenteriae* in *G. mellonella* surrogate model. (**a**) The survival of larvae was assessed at indicated times after injection of PBS (control) or 10^8^ of bacteria. The azithromycin and SFN alone or their combination were administrated 1 h after infection at a concentration indicated and development of the infection was observed during the next 96 h. (**b**) In vivo phagocytosis ratio of FITC-labelled bacteria. (**c**) Representative fluorescent microscopy images of bacteria phagocytosed by hemocytes (indicated by white arrows) from uninfected (PBS) and infected and SFN-treated larvae.

The larvae were then infected with bacteria and then injected with SFN and/or azithromycin (typically used antibiotic for treatment of bacterial gastroenteritis). For control, PBS injection was used. While the antibiotic alone has reduced insect mortality, a similar effect was observed after injection with SFN. In particular, when both azithromycin and SFN were used, mortality was the lowest, not exceeding 20% even after 96 h (Figure 5a). These results indicates that SFN alone has a significant anti-pathogenic activity, and in combination with azithromycin, this effect is even more pronounced. In the checkerboard tests conducted by us, SFN and azithromycin in combination showed an additive effect (FICI ≥ 1, data not shown). Interestingly, the ability of SFN to protect the larvae from developing a potentially lethal infection is related to activation of hemocytes. The percentage of activated hemocytes with phagocytosed bacteria was increased during SFN treatment (Figure 5b). Phagocytosis induces aggregation of hemocytes (Figure 5c), a process that is known to cause clearance of pathogens from the organism and the subsequent release of antimicrobial peptides [41,42].

The SFN action in promoting phagocytosis was widely investigated previously [43,44]. In our recent work, we have shown that this mechanism is involved in bacterial clearance in the *V. cholerae* infection [23]. The finding of therapeutic properties of SFN in the course of the *S. dysenteriae* infection of both cell lines and the animal model is a key step in developing the strategy to use the dietary phytoncides in therapy. Combining ITCs with antibiotics typically used to treat *Shigella* infections would possibly increase the antibacterial effect even more. Further thorough research with vertebrate models will provide a better insight into the prospective use of ITC in treating pathogenic infections in humans.

## 4. Conclusions

Bacterial infections comprise an increasing threat to public health, especially with the growing level of multiple antibiotic resistance among pathogens. Thus, a search for novel antibacterial compounds currently attracts attention of scientific institutions and health officials. In our work, we show the antibacterial effects of two plant secondary metabolites, PEITC and SFN, against *Shigella* strains. The established MIC indicates a potential for these compounds to be safely used. The mechanism underlying their antibacterial effect involves induction of the global stress response, the stringent response, also decreasing expression of the Shiga toxin genes. Bacterial growth inhibition and a decrease in toxicity, as demonstrated with the Vero and HeLa cell lines, indicate a therapeutical potential of ITCs. Most importantly, inhibition of the *S. dysenteriae* toxic effect against *Galleria mellonella* by SFN alone and in combination with azithromycin provides an important step in the prospective use of the plant-derived compounds to treat bacterial infections.

## Figures and Tables

**Table 1:** Susceptibility of *Shigella* species to PEITC and SFN.

Strain	MIC (MBC) [mM]	
	PEITC	SFN
*S. dysenteriae* SZ2855/97 Japan	0.50 (1.00)	0.25 (0.50)
*S. dysenteriae* Bonn/76	0.25 (0.25)	0.06 (0.125)
*S. dysenteriae* 156-36/88 Osaka	0.50 (0.50)	0.25 (0.50)
*S. sonnei*	1.00 (2.00)	2.00 (2.00)
*S. flexneri*	4.00 (ND ^1^)	4.00 (ND)

^1^ not determined.

## Data Availability

Not applicable.
